# Minimal clinically important differences for the EQ-5D and QWB-SA in Post-traumatic Stress Disorder (PTSD): results from a Doubly Randomized Preference Trial (DRPT)

**DOI:** 10.1186/1477-7525-11-59

**Published:** 2013-04-12

**Authors:** Quang A Le, Jason N Doctor, Lori A Zoellner, Norah C Feeny

**Affiliations:** 1 Department of Pharmacy Administration and Practice, Western University of Health Sciences, 309 E. Second Street, Pomona, CA 91766-1854, USA; 2 Department of Clinical Pharmacy, Pharmaceutical Economics and Policy/Leonard D. Schaeffer Center for Health Policy and Economics, University of Southern California, 3335 S. Figueroa Street, Unit A, Los Angeles, CA, 90089-7273, USA; 3 Department of Psychology/Center for Anxiety and Traumatic Stress, University of Washington, Box 351525, Seattle, WA, 98195-1525, USA; 4 Department of Psychological Sciences, Case Western Reserve University, 10900 Euclid Avenue, Cleveland, OH, 44106-7123, USA

**Keywords:** EQ-5D, QWB-SA, Minimal clinically important difference, PTSD, Doubly randomized preference trial, Prolonged exposure therapy, Sertraline

## Abstract

**Objective:**

To determine the minimal clinically important difference (MCID) for the health-utility measures EuroQol-5 dimensions (EQ-5D) and Quality of Well Being Self-Administered (QWB-SA) Scale in PTSD patients.

**Research design and methods:**

Two hundred patients aged 18 to 65 years with PTSD enrolled in a doubly randomized preference trial (DRPT) examining the treatment and treatment-preference effects between cognitive behavioral therapy and pharmacotherapy with sertraline and completed the EQ-5D and QWB-SA at baseline and 10-week post-treatment. The anchor-based methods utilized a Clinical Global Impression-Improvement (CGI-I) and Clinical Global Impression-Severity. We regressed the changes in EQ-5D and QWB-SA scores on changes in the anchors using ordinary least squares regression. The slopes (beta coefficients) were the rates of change in the anchors as functions of change in EQ-5D and QWB, which represent our estimates of MCID. In addition, we performed receiver operating characteristic (ROC) curve analysis to examine the relationship between the changes in EQ-5D and QWB-SA scores and treatment-response status. The MCIDs were estimated from the ROC curve where they best discriminate between treatment responders and non-responders. The distribution-based methods used small to moderate effect size in terms of 0.2 and 0.5 of standard deviation of the pre-treatment EQ-5D and QWB-SA scores.

**Results:**

The anchor-based methods estimated the MCID ranges of 0.05 to 0.08 for the EQ-5D and 0.03 to 0.05 for the QWB. The MCID ranges were higher with the distribution-based methods, ranging from 0.04 to 0.10 for the EQ-5D and 0.02 to 0.05 for the QWB-SA.

**Conclusions:**

The established MCID ranges of EQ-5D and QWB-SA can be a useful tool in assessing meaningful changes in patient’s quality of life for researchers and clinicians, and assisting health-policy makers to make informing decision in mental health treatment.

**Clinical trial registration:**

Clinicaltrials.gov; Identifier: NCT00127673.

## Introduction

Posttraumatic stress disorder (PTSD) is a chronic and debilitating condition, with lifetime prevalence rates ranging from 8%–14% of the US population [1]. Moreover, given recent estimates of PTSD among Operation Iraqi Freedom (OIF) and Operation Enduring Freedom (OEF) veterans, there is an unprecedented need for empirically-supported PTSD treatment for military personnel and veterans [2]. PTSD is associated with poor quality of life in multiple health domains [3-5] and also has a huge financial burden [5]. Greenberg and colleagues (1999) [6] reported that through work impairment, hospitalization, and health visits, PTSD was more costly than any other anxiety disorder. Among the 1.64 million veterans returning from OEF and OIF, it is estimated that approximately 300,000 individuals currently suffer from PTSD or major depression, potentially costing $4.0 to $6.2 billion in a two-year time frame [7]. These considerations highlight the substantial impact of PTSD and the need for reliable and valid measures of improved clinical outcomes.

Clinically, the PTSD Symptom Scale-Interview (PSS-I) [8], PTSD Checklist (PCL) [9], and Clinician-Administered PTSD Scale (CAPS) [10] have been the most commonly used measures for assessing symptomatic improvement/deterioration in clinical trials. In addition, patient-reported outcome (PRO) instruments have been increasingly utilized to supplement to the clinical measures and provide additional information on other health-related quality of life (HRQOL) domains (mobility, self-care, usual activities, pain/discomfort, social, emotional, and physical functions) as well as health utilities [5,11-14]. For example, to justify the cost of a new intervention in PTSD, health-policy makers would need to determine not only whether the new intervention provides significantly clinical improvement but also whether the new intervention is cost-effective as compared to the current standard treatment. Incorporating generic health-utility measures such as the EQ-5D, QWB-SA, Health Utility Index Mark 3 (HUI3), or Short Form-6 dimensions (SF-6D) can allow comparisons of burden of disease across health conditions as well as the quality-adjusted live years (QALYs), a HRQOL measure used to evaluate the cost-effectiveness for healthcare interventions. Nevertheless, interpretation of a change in HRQOL score from pre- to post-treatment can be confusing to clinicians and other health professionals due to their unfamiliarity with the PRO instruments. In contrast, repeated experience and familiarity with clinical measures such as Body Mass Index (BMI) or blood pressure allow health professionals to make meaningful interpretation of the measures [15]. Thus, by placing the magnitude of change in HRQOL score corresponding to a minimal clinically important difference would be helpful and meaningful for health professionals, patients, health-policy makers as well as other stakeholders [15].

In general, the minimal clinically important difference (MCID) of a PRO instrument is defined as smallest change in a PRO measure that is linked to a clinically relevant difference or change. In other words, MCID is the smallest difference that patients perceived as beneficial or harmful and that would result in a change in treatment [16]. There are two broad methods for estimating the MCID of a PRO instrument: (1) anchor-based methods, which link changes in HRQOL scores to external indicators either clinical or patient-based such as laboratory or physiological measures, and clinician or patient ratings; and (2) distribution-based methods, which estimate MCIDs using small to medium effect sizes based on the distribution of HRQOL scores in a relevant sample [17]. Nevertheless, since no single anchor is ideal and no single method is perfect, it is recommended that multiple approaches from both anchor- and distribution-based methods should be used to estimate the MCID for a PRO instrument [17].

Empirical work on MCIDs for the EQ-5D or QWB-SA has been done on several conditions [15,18-23]; however it has not performed in mental health disorders, particularly in PTSD. In the current study, we estimated and compared the smallest changes in HRQOL utility scores of EQ-5D and QWB-SA that can be regarded as clinically important in PTSD patients using multiple anchor- and distribution-based approaches.

## Patients and methods

**Figure 1:**
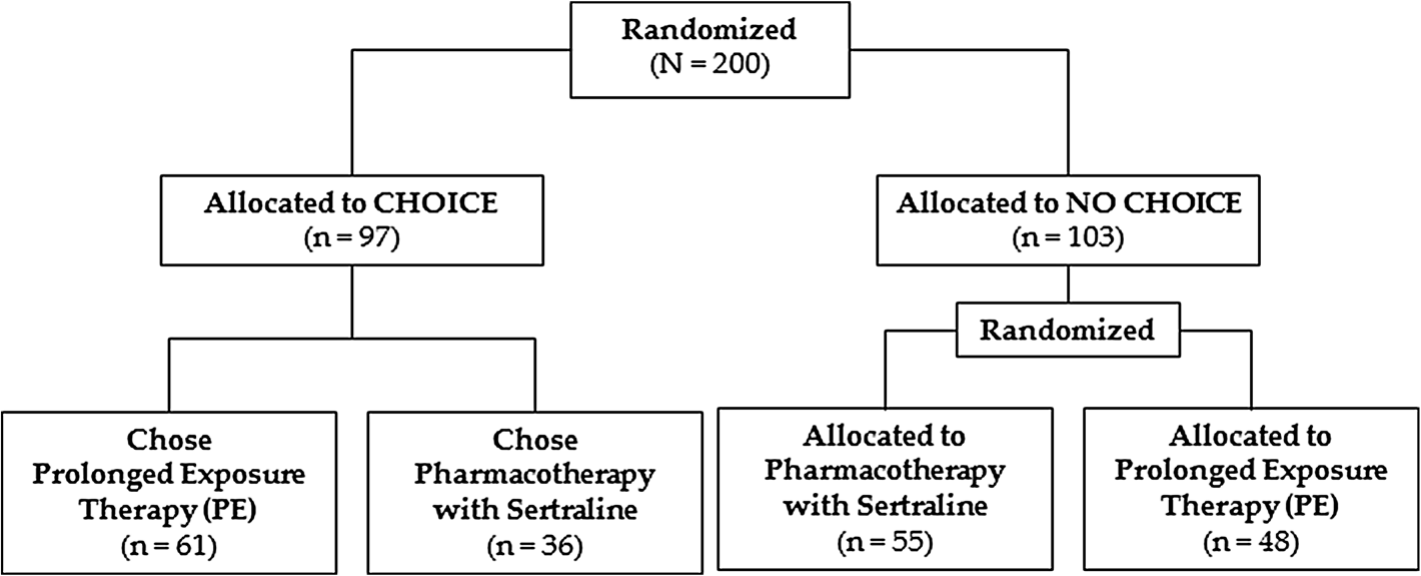
The Optimizing PTSD Treatment (OPT) Trial.

Data for analysis were from the Optimizing PTSD Treatment (OPT) trial (Clinicaltrials.gov; Identifier: NCT00127673). The OPT trial is a hybrid efficacy-effectiveness trial designed to better understand personalized PTSD treatment in two clinics at University of Washington, Seattle, WA, and Case Western Reserve University, Cleveland, OH. The OPT trial included patients between the age of 18 and 65 years, who were currently diagnosed with primary PTSD based on DSM-IV criteria, with a minimum duration of 12 weeks since the traumatic event and diagnosed using the PTSD Symptom Scale-Interview Version (PSS-I) [8]. Patients were excluded from the trial if they had one of the following: A current diagnosis of schizophrenia or delusional disorder; medically unstable bipolar disorder, depression with psychotic features, or depression severe enough to require immediate psychiatric treatment (e.g. actively suicidal); a current diagnosis of alcohol or substance dependence in the previous three months; an ongoing intimate relationship with perpetrator (in assault cases); unwillingness to discontinue current trauma-focused psychotherapy or anti-depressant medication (depending on the assigned treatment arm) or discontinuation was not medically advisable; either previous nonresponse to prolonged exposure or sertraline; and medical contraindication for the initiation of medication (e.g. pregnancy or lactation). Patients were randomly assigned to either choice or no choice treatment conditions, using a doubly randomized preference trial design (DRPT). In the choice condition, patients chose treatment between prolonged exposure therapy (PE) and pharmacotherapy with sertraline (SER). In the no-choice condition, patients were randomly assigned to either PE or pharmacotherapy (Figure 1). Patients received 10 weeks of acute treatment. Clinical and HRQOL measures were obtained from all willing patients at pre- and post-treatment, and at 3-, 6-, and 12-month follow-up.

### HRQOL health-utility instruments

The EQ-5D is a five-item self-administered questionnaire and one of the most widely used generic preference-based measures for estimating health utilities. The measure has 5 health domains (mobility, self-care, usual activities, pain/discomfort, and anxiety/depression), each with three response-levels: no problems, some problems, and severe problems [24]. The scoring system of EQ-5D used in this study was based on the U.S. population-based EQ-5D [25] ranging from -0.11 (all five ED-5D health domains reported extreme problems) to 1 or perfect health (no problems at all five EQ-5D domains), in which zero means dead and negative utility scores represent health states worse than dead.

The QWB-SA is also a common generic preference-based HRQOL measuring health utilities. Overall, the QWB-SA includes five parts: (1) Part I asks about 19 chronic symptoms or problems (yes/no question format), 25 acute physical symptoms and 11 mental health symptoms over the last 3 days (in the format of whether the symptom occurs “yesterday,” “2 days ago,” and/or “3 days ago”); (2) Part II uses a similar format but asks about self-care; (3) Part III asks about mobility; (4) Part IV ask about physical functioning; and (5) Part V asks about social activities. In all, the domain scores are combined into a single index score ranging from 0.09 (lowest possible health state) to 1 for perfect health, with zero means dead [26].

### Anchors

The Clinical Global Impression (CGI) is a brief clinician-rated instrument assessing: (1) *severity of illness* (CGI-S) using a 7-point Likert scale: 1 or “normal, not mentally ill,” 2 or “borderline mentally ill,” 3 or “mildly mentally ill,” 4 or “moderately mentally ill,” 5 or “markedly mentally ill,” 6 or “severely mentally ill,” and 7 or “among the most extremely mentally ill;” and (2) *global improvement or change* (CGI-I) also using a 7-point Likert scale: 1 or “very much improved,” 2 or “much improved,” 3 or “minimally improved,” 4 or “no change,” 5 or “slightly worse,” 6 or “much worse,” and 7 or “very much worse [27].

In addition to CGI-S and CGI-I, we also selected the PTSD Symptom Scale-Interview (PSS-I) [8] as our third anchor. Classification of treatment responder or non-responder at 10-week post-treatment was assessed using the PSS-I and CGI-I. The 17-item PSS-I uses DSM-IV symptom criteria; and each symptom is rated on a 0 (not at all) to 3 (5 or more times per week/very much) scale of frequency and/or severity [7]. The absolute cutoff scores of 23 or less on the PSS-I and 3 or lower on the CGI-I define the clinically meaningful improvement [28-30].

### Statistical analysis

To be included in this analysis, a patient had to baseline or pre-treatment and a follow-up assessment of EQ-5D, QWB-SA, CGI-S, CGI-I, and PSS-I. For patients who had multiple follow-up visits, the current analysis included the first follow-up assessment on which all measures completed. All analyses in the study were conducted using Stata release 12.0 (Stata Corp LP, College Station, TX, USA).

#### Anchor-based approach

Correlation coefficients between changes in EQ-5D scores and changes in anchor measures were determined to confirm the usefulness of the anchors. A correlation coefficient of 0.30 or more is needed in order to be considered a good anchor [17].

For the CGI-I anchor, we grouped “very much improved” with “very much worse,” “much improved” with “much worse,” and “minimally improved” with “slightly worse;” and those on the worsening side of the scale, the sign of the change in HRQOL health-utility scores is reversed, i.e. negative sign to positive and vice versa. We regressed the changes of the EQ-5D and QWB-SA scores on the transformed CGI-I using ordinary least squares method. The slopes (beta coefficients of the regression lines) were the rates of change in the anchor CGI-I as functions of change in EQ-5D and QWB-SA scores, which represented the estimates of the MCID. This method helped to prevent few worsening responses that may adversely affect the slope of the regression line; thus the estimated MCIDs were more stable and applicable to the entire scores of the EQ-5D and QWB-SA as opposed to separate the scores into worsening and improvement [31]. For the CGI-S anchor, to estimate the MCIDs, we simply regressed the changes of the EQ-5D and QWB-SA scores on the change of CGI-S between pre-treatment and follow-up visit.

In our second anchor-based approach, we analyzed the relationship between the changes in EQ-5D and QWB-SA scores and the treatment response status using receiver operating curve (ROC) analysis to estimate the MCIDs. The ROC curves were constructed by plotting the sensitivity (true-positive rate) against the one minus specificity (false-positive rate) at different cut-off points in the continuous HRQOL score changes that distinguished treatment responder and non-responder. The area under the ROC cure (AUC) can be interpreted as the probability of correctly discriminating between the treatment responder and non-responder [32-35]. The AUC ranges from 0.5 (corresponds to no discriminatory ability, i.e. random responding as with a coin flip to determine treatment-response status) to 1.0 (corresponds to perfect discriminatory ability, i.e. perfect prediction). Using ROC curve analysis, the MCIDs were determined based on the optimal cut-off points for the changes in HRQOL scores which maximized the sensitivity and specificity, i.e. point that best discriminated between patients who were treatment responders and those who did not respond to treatment [34,35].

#### Distribution-based approach

For distribution-based approach, the MCIDs can be estimated as one half the standard deviations of the pre-treatment EQ-5D and QWB-SA scores. The one half the standard deviation at baseline of a HRQOL measure (or moderate effect size) has been linked to establish the MCID and used widely in literature [36]. Alternatively, a small effect size in terms of 0.2 the standard deviations at pre-treatment EQ-5D and QWB-SA scores were also utilized [37].

## Results

Figure 1 provides the consort diagram for the OPT trial’s doubly randomized preference design. Two hundred confirmed PTSD patients were first randomly assigned to the choice (n = 97) and no-choice (n = 103) arms. In the choice arm, 67 patients chose prolonged exposure therapy while 36 patients chose pharmacotherapy with sertraline. In the no-choice arm, patients were randomized again to PE (n = 55) and SER (n = 48). Similar demographic and clinical characteristics were observed across the four arms of the trial (Table 1). In the overall sample, patients were between moderately and markedly mentally ill (mean CGS-S = 4.6), primarily female (76%), middle aged (37.5 years), and did not have 4-year college degree. Additionally, a wide range of EQ-5D (0.17 to 1.0) and QWB-SA scores (0.22 to 0.86) were observed. The overall mean of EQ-5D score was larger than the mean QWB-SA score (0.63 ± 0.19 vs. 0.57 ± 0.11) (Table 1).

**Table 1:** Baseline demographic and clinical characteristics

	**NO**-**CHOICE **(**RANDOMIZATION**)				**CHOICE**		**TOTAL**
	**PE**	**SER**	**Subtotal**	**PE**	**SER**	**Subtotal**	
Number of Patients (%)	55	48	103	61	36	97	200
Age in years, mean (SD)	36.2 (11.4)	38.9 (11.3)	37.5 (11.4)	37.1 (11.3)	38.3 (11.4)	37.5 (11.3)	37.5 (11.3)
Gender (%)							
Female	78.2%	79.2%	78.6%	75.4%	69.4%	73.2%	76.0%
Male	21.8%	20.8%	21.4%	24.6%	30.6%	26.8%	24.0%
Education (College Educated) (%)	38.2%	23.0%	31.1%	42.6%	22.2%	35.1%	33.0%
PTSD Severity (PSS-I), mean (SD)	29.7 (7.1)	29.6 (6.3)	29.7 (6.7)	29.1 (6.8)	30.0 (6.7)	29.5 (6.7)	29.6 (6.7)
Re-experiencing, mean (SD)	7.8 (2.7)	7.5 (3.0)	7.7 (2.9)	7.3 (2.7)	7.5 (2.8)	7.4 (2.7)	7.5 (2.8)
Avoidance, mean (SD)	12.3 (3.8)	11.9 (3.4)	12.1 (3.6)	12.2 (3.1)	12.4 (3.1)	12.3 (3.1)	12.2 (3.3)
Hyperarousal, mean (SD)	9.6 (3.3)	10.2 (2.5)	9.9 (2.9)	9.6 (2.9)	10.1 (3.1)	9.8 (3.0)	9.8 (3.0)
CGI-S, mean (SD)	4.6 (1.0)	4.5 (0.9)	4.6 (1.0)	4.6 (1.1)	4.4 (0.9)	4.5 (1.0)	4.6 (1.0)
EQ-5D, mean (SD)	0.67 (0.18)	0.60 (0.21)	0.61 (0.20)	0.65 (0.17)	0.56 (0.19)	0.62 (0.18)	0.63 (0.19)
QWB-SA, mean (SD)	0.58 (0.11)	0.57 (0.10)	0.57 (0.11)	0.56 (0.10)	0.59 (0.11)	0.57 (0.10)	0.57 (0.11)

SER, Pharmacotherapy with sertraline; PE, Prolonged Exposure Therapy; PTSD, Post-traumatic Stress Disorder; PSS-I, PTSD Symptom Scale-Interview; CGI-S, Clinical Global Impression-Severity; EQ-5D, EuroQol Group-5 Dimensions; QWB-SA, Quality of Well-Being-Self Administration.

Table 2 shows the correlation coefficients between the HRQOL health-utility measures (EQ-5D and QWB-SA) and the clinical anchors (CGI-I, CGI-S, PSS-I, and treatment response status). Overall, all the clinical anchors were strongly correlated with the HRQOL health-utility measures (Spearman’s Rho ranged from 0.35 to 0.44 with significant alpha level < 0.001); thus were considered appropriate anchors.

**Table 2:** **Correlation coefficients between the HRQOL health**-**utility measures** (**EQ**-**5D and QWB**-**SA**) **and the clinical anchors** (**CGI**-**I**, **CGI**-**S**, **PSS**-**I**, **and treatment response status**)

	**ANCHORS**			
**HRQOL health**-**utility instrument**	**CGI**-**I**	**CGI**-**S**	**PSS**-**I**	**Treatment response status**
EQ-5D	-0.35*	0.37*	0.44*	0.38*
QWB-SA	-0.41*	0.39*	0.43*	0.38*

HRQOL, Health-Related Quality of Life; CGI-I, Clinical Global Impression-Improvement; CGI-S, Clinical Global Impression-Severity; PTSD, Post-traumatic Stress Disorder; PSS-I, PTSD Symptom Scale-Interview; EQ-5D, EuroQol Group-5 Dimensions; QWB-SA, Quality of Well-Being-Self Administration.

* Statistical Significance at α < 0.001.

One hundred and fifty patients had all assessment of EQ-5D, QWB-SA, CGI-S, CGI-I, and PSS-I at baseline and at least one follow-up visit. Table 3 gives the mean changes of the HRQOL health-utility scores and clinical anchors between pre-treatment and follow-up visit. All the changes in scores had medium or large effect sizes. Of these 155 patients, 130 patients (83.9%) responded to one of the therapies.

**Table 3:** **Changes in HRQOL health**-**utility scores and clinical anchors between pre**-**treatment and follow**-**up**

**Measures**	**N**	**Mean (SD)**	**Effect size**
CGI-S	155	-2.6 (1.5)	2.06
CGI-I*	155	1.7 (1.1)	N/A
PSS-I	155	17.9 (10.8)	2.03
Treatment Response†	155	130 (83.9%)	N/A
EQ-5D	155	0.15 (0.22)	0.77
QWB-SA	155	0.09 (0.15)	0.64

HRQOL, Health-Related Quality of Life; SD, Standard Deviation; CGI-I, Clinical Global Impression-Improvement; CGI-S, Clinical Global Impression-Severity; PTSD, Post-traumatic Stress Disorder; PSS-I, PTSD Symptom Scale-Interview; EQ-5D, EuroQol Group-5 Dimensions; QWB-SA, Quality of Well-Being-Self Administration.

* Note that CGI-I was only given at post-treatment and thereafter.

† Treatment response was measured as number (n) and percent (%) of patients who responded to treatment.

Table 4 summarizes the MCID estimates for the EQ-5D and QWB-SA using multiple approaches from both anchor- and distribution-based methods. The EQ-5D MCID ranges from 0.05 to 0.08 using the anchor-based approach and from 0.04 to 0.10 using distribution-based approach. For the QWB-SA, using the anchor- and distribution-based approaches resulted in the ranges of MCID values from 0.02 to 0.05.

**Table 4:** **Estimated Minimal Clinically Important Differences** (**MCIDs**) **and their 95**% **Confidence Intervals** (**CIs**) **for EQ**-**5D and QWB**-**SA using both anchor**- **and distribution**-**based approaches**

**ANCHOR**-**BASED APPROACH**				**DISTRIBUTION**-**BASED APPROACH**	
**HRQOL health**-**utility**	**CGI**-I*	**CGI**-S*	**Treatment response status†**	**0**.**2 SD**	**0**.**5 SD**
EQ-5D	0.08 (0.04–0.11)	0.05 (0.03–0.07)	0.05	0.04	0.10
QWB-SA	0.05 (0.03–0.08)	0.03 (0.02–0.05)	0.03	0.02	0.05

HRQOL, Health-Related Quality of Life; CGI-I, Clinical Global Impression-Improvement; CGI-S, Clinical Global Impression-Severity; SD, Standard Deviation at baseline or pre-treatment; EQ-5D, EuroQol Group-5 Dimensions; QWB-SA, Quality of Well-Being-Self Administration.

**Figure 2:**
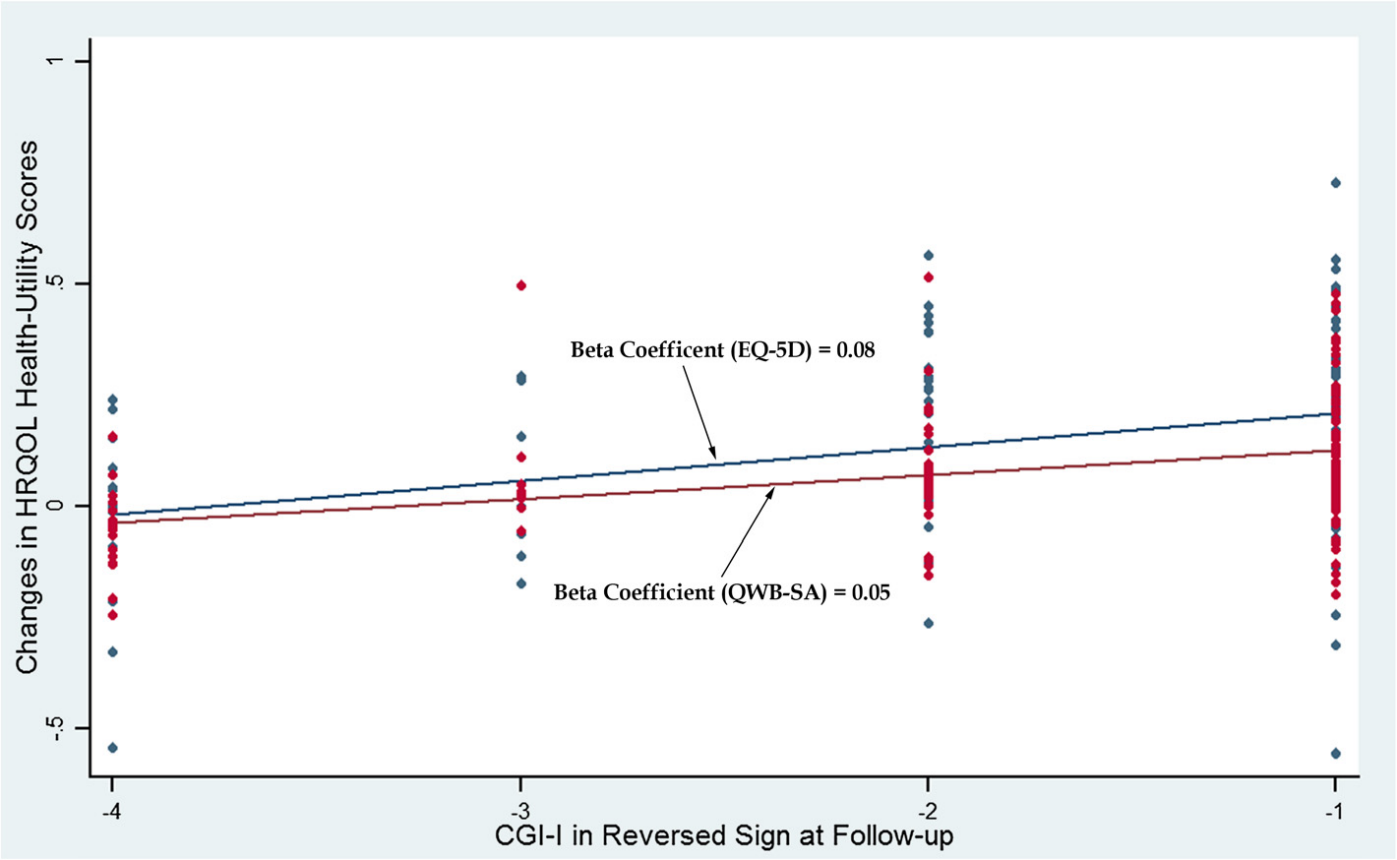
EQ-5D and QWB-SA versus CGI-I-Scatter Plot and Regression Line.

**Figure 3:**
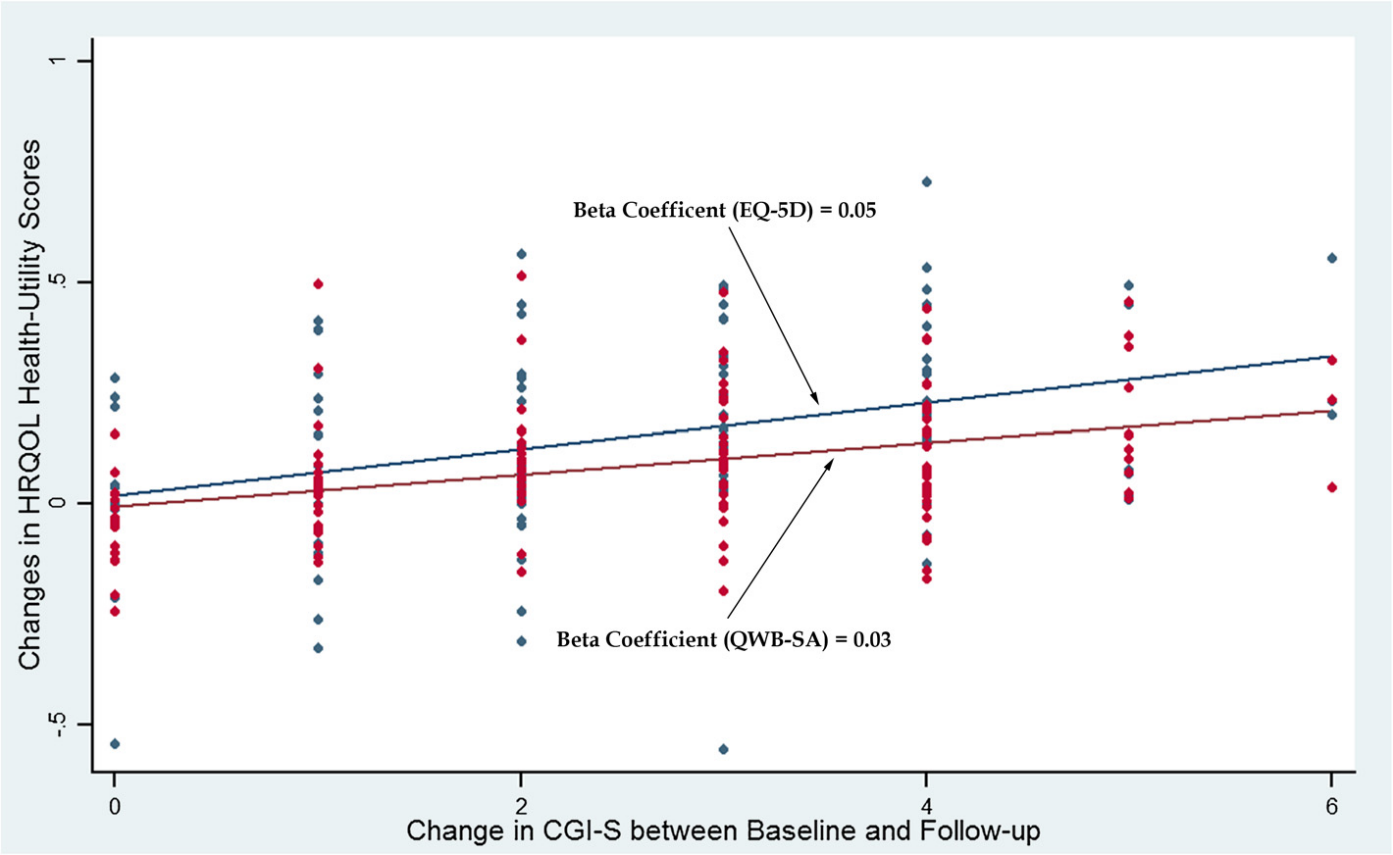
EQ-5D and QWB-SA versus CGI-S-Scatter Plot and Regression Line.

* Anchor-based approach using ordinary least squares regression method (also refer to Figures 2 and 3).

**Figure 4:**
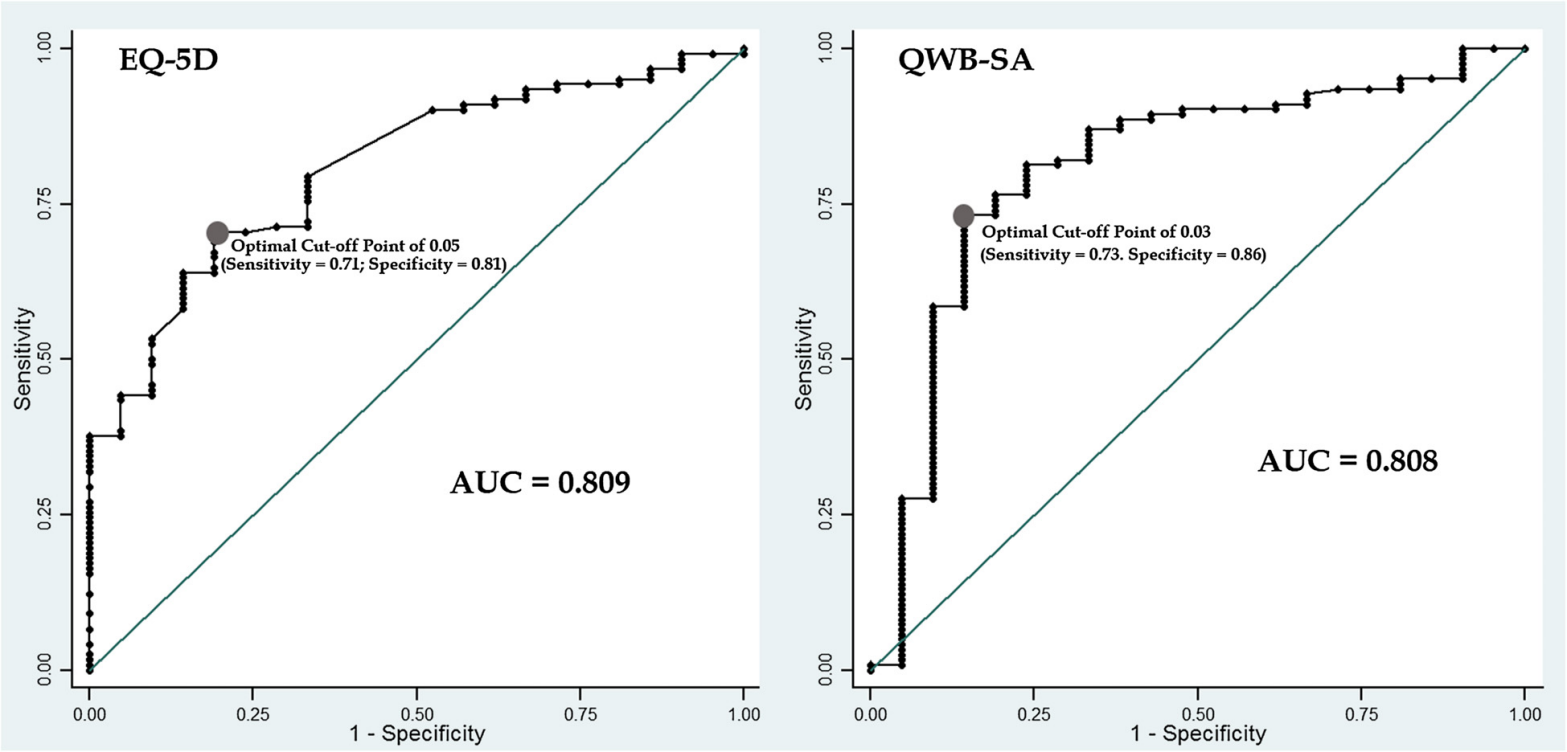
ROC Curves of EQ-5D and QWB-SA with Optimal Cut-off Points.

† Anchor-based approach using ROC curve analysis (also refer to Figure 4).

Figures 2 and 3 demonstrate the regression lines of the changes of the EQ-5D and QWB-SA scores on the transformed CGI-I and CGI-S. The beta coefficients of the regression lines (the slopes) indicate the estimates of the MCIDs (0.05 to 0.08 for the EQ-5D and 0.03 to 0.05 for the QWB-SA). Figure 4 shows the ROC curves with the optimal cut-off points represented the MCIDs for the EQ-5D and QWB-SA, respectively. For the EQ-5D (AUC = 0.809; 95% CI: 0.721–0.897), at the optimal cut-off point of 0.05 it maximized the sensitivity (0.71) and specificity (0.81). For the QWB-SA (AUC = 0.808; 95% CI: 0.696–0.920), the optimal cut-off point of 0.03 resulted in maximized sensitivity (0.73) and specificity (0.86).

## Discussion

Understanding changes in scores and how to interpret the changes are critical in the field of HRQOL measurement. Because there is no single gold-standard method for estimating MCID, multiple methods from both anchor- and distribution-based approaches and triangulation of all the methods to establish a possible range of MCID are recommended [17]. Using data from a doubly randomized preference trial in post-traumatic stress disorder patients (the OPT trial), our analysis suggests that the plausible range of MCID values for the HRQOL health-utility EQ-5D and QWB-SA in the population of PTSD patients were between 0.04 and 0.10, and 0.02 to 0.05, respectively. Empirical works on MCIDs for the EQ-5D or QWB-SA has been done on several disease states and were ranged from -0.01 to 0.14 [15,18-21]. However, those MCID estimates for the EQ-5D were based on the U.K. scoring algorithm or EQ-5D VAS instead of the U.S. scoring method used in the current study. Two studies using the U.S. population-based scoring model reported similar range of MCID values between 0.07 and 0.09 for the EQ-5D utility [18,20]. For the QWB-SA, our range of MCID values was consistent with previous studies [22,23].

The clinical anchors (CGI-I, CGI-S, and PSS-I for classifying treatment response status) used in our analysis were most appropriate as they were highly clinically relevant and strongly correlated with the HRQOL health-utility EQ-5D and QWB-SA. In addition, the anchor-based approach utilized well-established methods (OLS regression and ROC curve analysis) to estimate the MCIDs and produced rather similar results even if with different anchors used. The AUCs resulted from ROC analysis were rather large for both EQ-5D and QWB-SA indicating that the HRQOL health-utility measures had great ability to discriminate correctly treatment responders from non-responders. Although multiple methods are necessary to estimate a range of MCID values, Revicki and colleagues (2008) [17] further recommended that results from the anchor-based approach have the most weight due to their clinical advantages over the distribution-based approach. That is, it is more likely that the ranges of MCID values in the population of PTSD patients would be between 0.05 to 0.08 and 0.03 to 0.05 for the EQ-5D and QWB-SA, respectively.

Both EQ-5D and QWB-SA are assumed to measure the same underlying construct of overall HRQOL in terms of health utility. The primary use of HRQOL health-utility measures is to calculate the quality-adjusted life year (QALY), a function of both quantity and quality of life, which is used in health economic evaluations and decision models to help health policy makers to allocate resources effectively. Therefore, it is important to establish their MCIDs and then compare them between the EQ-5D and QWB-SA. Our results showed that the plausible range of MCID values for the EQ-5D was almost twice that of the range for the QWB-SA. It was more likely because the two HRQOL health-utility instruments: (1) measure different health state descriptive systems thus different number of possible health states (243 possible health states for the EQ-5D versus 945 for the QWB-SA), (2) assess preferences for the multiple health states using different methods, i.e. time-trade off method used for the EQ-5D and rating-scale for the QWB-SA, and (3) use different scoring functions.

There were, however, some limitations in the current analysis. First, we did not apply multiple imputation methods for the missing data. Instead, we assumed that any missing assessments of the clinical anchors and HRQOL health-utility measures were missing completely at random (MCAR), meaning that our results would be similar whether or not there were missing data. Secondly, as there were very few worsening cases, the anchor-based methods focused mainly on the responses of those who were clinically improved rather than those worsened. Future work to assess the MCIDs for those who are clinically worsened is in need. Nevertheless, more than often the MCID is used in the context of a treatment effect, thus the MCID results in our study can still be applied to detect minimal clinically improvement in score changes. Finally, in our study, CGI-I questions were given to patients at 10-week post-treatment. The main limitation of using anchor-based approach that relies on global ratings is that these retrospective ratings are potentially susceptible to recall bias. As discussed above, it is important to estimate a range of MCID values from several different methods rather than a point estimate.

Our analysis to determine the plausible ranges of MCID values for the EQ-5D and QWB-SA followed the recommendations by Revicki and colleagues (2008) [17]: longitudinal data were obtained from the clinical trial, multiple anchors were used and they were highly clinically relevant and strongly correlated with the HRQOL instruments, methodologically sound methods utilizing OLS regression and ROC curve analysis were applied in the anchor-based approach, and triangulation of multiple methods using both anchor- and distribution-based approaches to produce plausible ranges of MCID values.

## Conclusion

To our knowledge, this analysis is the first attempt to use multiple anchors-based approach as well as distribution-based approach to determine and compare the MCID ranges for the EQ-5D and QWB-SA in the population of PTSD patients. The information can be helpful in interpreting the EQ-5D and QWB-SA scores as well as in planning new trials when estimating power and sample sizes [15]. Furthermore, the established MCID ranges of EQ-5D and QWB-SA can be a useful tool in assessing meaningful changes in patient’s quality of life for researchers and clinicians, and assisting health-policy makers to make informing decision in mental health treatment.

## Consents

Written informed consent was obtained from the patient for publication of this report and any accompanying images.

## Competing interests

The authors declare that they have no competing interests.

## Authors’ contributions

LZ and NF were co-PIs, designed, coordinated, and collected data for the clinical trial. QL and JD performed statistical analyses, interpreted results, and drafted the manuscript of this study. All authors read and approved the final manuscript.
